# How does digital literacy affect the health status of senior citizens? Micro-level evidence from the CFPS data

**DOI:** 10.1186/s12913-025-12314-7

**Published:** 2025-01-27

**Authors:** Xiaoyi Chen, Nian Wang

**Affiliations:** 1 College of Digital Economics, Nanning University, Nanning, 530000 China; 2 China-ASEAN Institute of Statistics, Guangxi University of Finance and Economics, Mingxiu West Road 100, Nanning, 530003 China

**Keywords:** Digital literacy, Health status, Senior citizens, Social support, Mediation mechanism

## Abstract

**Background:**

The popularization of the Internet and digital technology has called for higher digital literacy among citizens, especially the elderly. However, most existing studies didn’t measure digital literacy at the micro level, and the impact mechanism has rarely been discussed. The purpose of this study is to clarify whether and how digital literacy affects the health status of senior citizens.

**Methods:**

The data used in this study are collected from the China Family Panel Studies (CFPS) from three periods: 2016, 2018, and 2020. The balanced panel data comprised 7836 samples, with 2612 samples per year. Based on the CFPS data, this study constructs a balanced panel and employs a two-way fixed-effects model for the estimation. The instrumental variable (IV) method is employed for tackling the endogenous problems. Next, the mediation effect model is applied to identify the influencing mechanism.

**Results:**

First, digital literacy can improve the health status of senior citizens. This result remains valid after introducing lagged explanatory variables and addressing the endogeneity issues. Second, social support acts as a partial mediator in the relationship between digital literacy and the health status of senior citizens. Third, the heterogeneity analysis reveals that the effect of digital literacy on the health status of senior citizens varies across age groups, urban–rural types, and education levels.

**Conclusions:**

This study examines the impact of digital literacy on the health status of senior citizens at the micro-level and identifies the mediation mechanism. The results enhance our understanding of the positive effects of digitalization on aging society and offer useful insights for the government in formulating more targeted active aging strategies.

## Introduction

Health status serves as a crucial indicator for measuring the well-being of senior citizens [1–3]. As the World Health Organization (WHO) emphasizes, improving the health status of the elderly not only enhances their quality of life, but also helps achieve better overall societal benefits [4]. So far, researchers have demonstrated many influencing factors of the health status of the elderly, including micro-level factors such as habits [5], religious beliefs [6], and socio-economic status [7], as well as macro-level factors including environmental pollution [8], sociocultural aspects [9], income disparities [10], and the level of openness to external influences [11]. With the blossom of digital technologies, such as 5G and smart city, digitization has increasingly penetrated into our socio-economic life [12, 13], and the concept of "digital literacy" has emerged. In the earlier studies, digital literacy referred to the ability to read and understand multimedia content [14]. Eshet and Amichai expanded the definition of digital literacy, defining it as the essential life skills required for individuals to live, learn, and work with digital technologies under the current digital environment [15]. The United Nations Educational, Scientific and Cultural Organization (UNESCO) proposes that digital literacy should include device operation, information processing, communication and writing, content creation, security protection, problem-solving, and specific occupation-related fields [16]. Given the accelerating digitalization process and aging trend worldwide, it is of great research significance to explore whether the enhancement of digital literacy among the elderly has a significant impact on their health status.

**Fig. 1:**
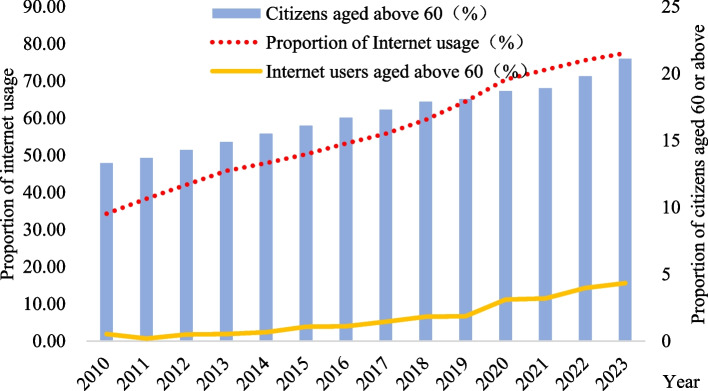
Internet usage by seniors and aging level in China

While the topic of aging and health is a global issue, developing countries face greater challenges in maintaining and improving the health status of the elderly due to the relatively backward medical technology compared to developed countries, as well as the immaturity of their healthcare systems. According to Frank et al., a country is considered to have entered an aging society when the proportion of its population aged 60 and above exceeds 10% [1]. As a member of the developing world, China has witnessed a rapid acceleration of aging over the past decade. Based on the data released by the National Bureau of Statistics,^1^ by 2023 (Fig. 1), citizens aged 60 or above accounted for 21.1% of the total population, an increase of nearly 8 percentage points compared to 2010. At the same time, the digitalization is experiencing rapid development. According to the Statistical Report on the Development of the Internet in China, from 2010 to 2023, China's Internet penetration rate increased by about 33%, and the proportion of Internet users aged over 60 raised from 1.9% to 15.6%. It can be seen that as China's Internet penetration rate continues to increase, the size of the elderly Internet user group is also expanding. Therefore, exploring the impact of digital literacy on the health status of the elderly based on Chinese samples and identifying the underlying mechanisms will not only contribute to improving the health status of the elderly in China, but also provide valuable insights for other developing countries in addressing the health issues of their aging populations.

Given this, our study attempts to explore the impact of digital literacy on the health of the elderly and identify the mechanism. This will not only facilitate healthy aging, but also provide statistical references on how "Digital China" drives the "active aging of the population".

According to the existing research [12, 13, 17], discussions regarding the impact of digitization on the health of senior citizens have primarily focused on the effects of digital technology development and internet usage. Many studies demonstrated that the utilization of digital technology can enhance the accessibility and convenience of high-quality healthcare services, thereby contributing to the maintenance of physical health among the elderly [17–20]. In addition to this, the application of digital technology in the financial sector can improve household medical utilization through means such as mobile payments and credit access [21]. Moreover, it enhances the accessibility of older people to convenient financial services and products, thereby contributing to the alleviation of financing constraints and strengthening the economic foundation for improving the health status of the elderly [22–24].

In terms of the influencing mechanism, some studies argue that income level serves as a critical factor. The positive correlation between health and income has been supported by extensive research [25–28]. At the same time, digital literacy can affect individual income at both the macro- and micro-levels. Regarding the macro-level, digital literacy enables people to grasp new business opportunities [29, 30], which can lead to economic development and, consequently, to income improvement [31]. At the micro-level, people's access to information has been broadened; thus, they have benefited from the development of digital technology. For example, the vigorous promotion of online education has greatly improved the labor skills and efficiency of workers and contributed to the increase in their income levels [32]. Some scholars also identified the usage of the Internet as one of the mechanisms by which digital literacy affects health status. A study by the Research Group of the Chinese Academy of Social Sciences found that the Internet has increased the rate of older people's communication with the outside world; it enhances their social links and shortens the social distance. The emergence of new media on the Internet has enriched the leisure life of the elderly and facilitated the provision of emotional value. Therefore, the use of the Internet by the elderly in terms of social and recreational activities has a positive impact on their physical and mental health. However, older people's commercial use of the Internet may have a negative impact on their physical and mental health, although this impact does not pass the statistical significance test [33].

The existing literature provides a rich discussion on the relationship between digital literacy and older people's health status [17–24, 29–33]. However, because they are confined by data limitations, the existing studies mainly focus on the macro-level, or simply employ Internet usage as the variable to measure digital literacy. Moreover, some of the research did not address the endogeneity problems caused by ignoring variables and reverse causation. With regard to this, this study aims to examine the impact of digital literacy on the health status of senior citizens at the micro-level by utilizing the multi-period Chinese Family Tracking Survey (CFPS) data from 2016 to 2020. The entropy method is used to measure the level of digital literacy of the elderly. Then, the two-way fixed-effects model is constructed to analyze the impact, and the instrumental variable method is adopted to alleviate the problems of reverse causation and omitted variables. Next, we introduce social support as the mediating variable to identify the influencing mechanism by which digital literacy affects the health status of senior citizens. Our results can provide useful insights for countries going through digitalization and aging society.

The marginal contributions of this study, compared to the existing literature [17–24, 29–33], lie in the following aspects. First, unlike previous studies that use a single indicator to characterize the level of digital literacy, this study employs multiple indicators with the entropy method to measure the digital literacy level of senior citizens, thereby reflecting the individual's digital literacy more comprehensively. Second, our study identifies the mediating mechanism of social support, broadening the research perspective on how digital literacy affects elderly health. Third, by conducting heterogeneous analysis across age groups, household registration, and education levels, our study offers valuable insights for the government in formulating more targeted active aging strategies.

The remaining sections are organized as follows. "Theoretical analysis and research hypotheses" discusses the theoretical analysis and research hypotheses. "Research design" provides the research design. "Empirical results and discussions" presents the empirical results and discussions. " Discussions" provides a summary with conclusions and policy implications.

## Theoretical analysis and research hypotheses

### The impact of digital literacy on the health status of senior citizens

Drawing upon the previous studies, it is clear that most research support the positive relationship between digital literacy and the health status of the elderly [29–33]. The first obvious explanation is that the increase in digital literacy facilitates the popularization of health knowledge, increasing the importance that older people attach to their health, establishing a more scientific outlook on health, and making them more willing to invest in their own health, thus helping to maintain their health status [34, 35]. Digitalization provides rich online medical resources, including online consultation and cyber doctors. Higher digital literacy enables older people to get access to online public health services, which contributes to the timely provision of medical guidance to older persons [36–38]. In addition, in the digital society, the leisure and entertainment functions provided by the Internet, such as online social communication and online games, can reduce the psychological loneliness of the elderly, and improve their sense of pleasure and attention, thus relieving psychological depression and preventing cognitive decline [39–41].

Based on the above, we propose Hypothesis 1:

- H1: Digital literacy levels significantly improve the health status of senior citizens.

### The mediating mechanism of digital literacy on the health status of senior citizens

The Social Support Theory [42] highlights the view that individuals derive both material and emotional support primarily from the social relationships they establish with others. This support enables them to cope with stress, challenges, and difficulties, thereby improving their health status [43]. Effective social support can assist the elderly in overcoming digital barriers and better embracing the digital society [44]. Seniors who receive more social support tend to have less stress and better health outcomes [45, 46].

The digital society, characterized by the prevalence of the Internet, breaks the constraints of time and space in communication. Individuals equipped with a certain level of digital literacy are capable of communicating with others using online messages and video calls, which extends the scope and frequency of social interactions and enhances social support. The social networks of the elderly population will no longer be confined to traditional circles of acquaintances [47, 48] since digital platforms bring together individuals with similar interests and hobbies. Additionally, when elderly gain assistance and support from children and grandchildren, it will strengthen the emotional bonds and provide psychological compensation. Moreover, online communication enhances social support and reduces the risk of depression [49, 50]. It is also worth noting that effective social support provided by family members plays a positive role in bridging the digital divide [51]. Furthermore, intergenerational interaction between parents and children can increase the initiative of seniors in acquiring digital knowledge and can enhance their ability and proficiency in using digital tools [52].

Based on the above, we propose Hypothesis 2:

- H2: Social support plays a mediating role in the relationship between digital literacy and the health status of senior citizens.

**Fig. 2:**
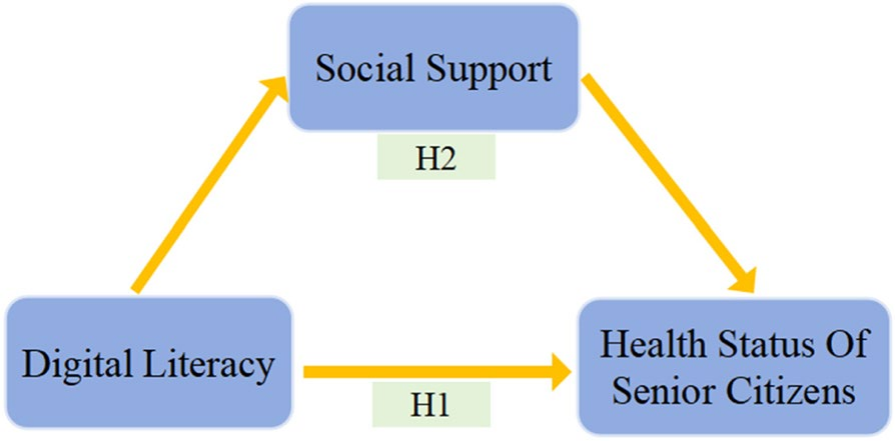
The influencing channel through which digital literacy affects the health status of senior citizens

The influencing channel is provided in Fig. 2.

## Research design

### Data and variable

#### Data

The data used in this study are collected from the China Family Panel Studies (CFPS). This survey started in 2010 and covers 25 provinces, municipalities, and autonomous regions in China; it has collected abundant data on income, consumption, health behaviors, education, and other aspects at the individual, family, and community levels. The dataset has high quality data and good representativeness, with an annual data tracking rate exceeding 80%.

Although the CFPS survey has collected data across five waves spanning from 2010 to 2020, the surveys conducted in 2010, 2012, and 2014 failed to address issues related to digital literacy properly. The questionnaires from 2016, 2018, and 2020 were refined by incorporating questions concerning senior citizens' use of mobile devices for internet access, as well as their engagement in learning, entertainment, and transactions through digital devices, which corresponds with our research objectives. More importantly, there were no changes in the phrasing of these questions, ensuring the continuity and stability of the key variables required for this study. Furthermore, since panel data, compared to cross-sectional data, provides more data points and control for individual differences that do not change over time, thereby enabling more accurate research conclusions, this study utilizes data from the CFPS surveys conducted in three waves, 2016, 2018, and 2020.

Given the large volume of micro-level data, we utilized Stata 15.0 to filter data that meet the research requirements, matching them annually based on sample IDs to construct panel data. Then, we employed descriptive statistical analysis and panel regression models to conduct the empirical analysis.

#### Variables

Dependent variable: the health status of senior citizens (health). The health status is an ordinal variable, with respondents answering about their own health condition. The variable is assigned a value of 1 when the answer is "unhealthy", a value of 2 when the answer is "average", a value of 3 when the answer is "relatively healthy", a value of 4 when the answer is "very healthy", and a value of 5 when the answer is "extremely healthy".

In accordance with the research objectives of this paper and the characteristics of the CFPS dataset, self-rated health is employed as a proxy for the health status of elderly individuals. The main reasons are as follow. Firstly, compared to macro-level measurements of overall health levels such as mortality rates and life expectancy, self-rated health offers a more precise measurement of individual health conditions in that it can reflect individual differences. Therefore, this indicator has been adopted by many existing studies [53–55]. Secondly, previous studies have confirmed the effectiveness of self-rated health in predicting mortality and anticipating functional impairments resulting from certain diseases [56, 57]. Thirdly, since conducting physical examinations on each sample to obtain accurate health data is impractical, self-rated health is the most feasible and practical proxy for the health status of elderly individuals among the indicators available in the CFPS database. Considering that self-rated health may introduce bias into the research results, other health indicators from the questionnaire are incorporated during the robustness checks.

Independent variable: digital literacy (digital). Digital transformation not only takes place at the national socio-economic and enterprise levels but also at the individual level. Unlike the digital economy at the macro-level or the digital transformation of enterprises at the micro-level, the individual's digital literacy is a combination of both macro-supply and micro-demand. It is mainly represented by the individual’s application of digital technologies, such as online payments, e-learning, and online shopping. Following the definition of digital literacy in the existing literature [15, 16, 52], this paper proposes that in the Chinese context, digital literacy refers to the digital application abilities of individuals to correctly and reasonably use digital tools and devices, gather digital resources to acquire new information and learn new knowledge, engage in social communication with others, and conduct business activities on digital platforms.

When it comes to measuring the digital literacy level, many studies simply use the single indicator method, which considers "whether the Internet is used" [58, 59]; this seems to be one-sided. To comprehensively measure the digital literacy level of senior citizens and avoid the one-sidedness of information reflected by a single indicator, this study constructs an index system of senior citizens’ digital literacy. Specifically, this paper categorizes digital literacy into four dimensions, digital tool usage literacy, digital learning literacy, digital entertainment literacy, and digital commerce literacy, as illustrated in Table 1. The digital literacy index for the elderly is calculated using the entropy weight method. The specific calculation process is given later.

**Table 1:** The index system of senior citizens’ digital literacy

dimension	variable
digital tool usage literacy	whether or not one uses mobile internet
	whether or not one uses a computer to access the internet
digital learning literacy	whether or not one engages in online learning
digital entertainment literacy	whether or not one watches short videos
digital commerce literacy	whether or not one engages in online shopping

Mediating variable: social support (issupport). In the CFPS questionnaire, social support is primarily represented by questions such as "How is your relationship with your children?", "How frequently do you contact your children?", "How often do you meet with your children?", "Do you think most people are helpful or selfish?", "How much do you trust your neighbors?", and "Do you have someone to take care of you when you are sick?". The response options range from 0 to 10, with higher values indicating stronger social support. The intensity of social support is calculated by summing up the scores of each response.

Instrumental variable: In order to address the endogeneity issue, this study introduces two instrumental variables. The first one is the product of the digital financial inclusion index (dfiindex) and the community average of digital literacy (mean_di), while the second one is the product of total postal and telecommunications business volume (tbtbusiness) and the community average of digital literacy (mean_di). The digital financial inclusion index (dfiindex) is published by the Institute of Digital Finance at Peking University; the total postal and telecommunications business volume by province (tbtbusiness) is collected from the National Bureau of Statistics; and the community average of digital literacy (mean_di) is the average digital literacy level of all elderly individuals within the community, excluding the individuals themselves.

Control variables: The health status of senior citizens is influenced by many factors. Variables representing individual characteristics, including gender (gender), age (age), education level (edu), Hukou status (hk), marital status (marry), pension insurance status (pension), and income status (income), are introduced to control for individual differences. Since lifestyle can also affect the health status of the elderly, this study also includes smoking habits (smoke), napping (noonbreak), and exercises (exercise) to control for personal lifestyle factors.

Table 2 displays the definitions of the variables.

**Table 2:** Definitions of variables

Variable type	Variable	Definition
Dependent variable	*health*	Ordinal variable. The respondents answer about their own health status, with a value range of 1–5, where a higher value represents better health status
Independent variable	*digital*	Continuous Variable, Calculated using the entropy method. The indicators include: whether or not one uses mobile internet, whether or not one uses a computer to access the internet, whether or not one engages in online shopping, whether or not one engages in online learning, and whether or not one watches short videos
Mediating variable	*issupport*	Ordinal variable. The value ranges from 3 to 35, with a higher value indicating a stronger intensity of social support
Instrumental variable	*iv1*	Continuous Variable. The product of *dfiindex* and *mean_di*
	*iv2*	Continuous Variable. The product of *tbtbusiness* and *mean_di*
Control variable	*age*	Continuous Variable
	*gender*	Dummy variable. A value of 0 indicates female, and 1 represents male
	*edu*	Ordinal variable. Below primary school = 0; Primary school = 6; Junior high school = 9; High school / vocational high school / technical secondary school = 12; Junior college = 15; Bachelor's degree = 16; Master's degree = 19; Doctoral degree = 22
	*hk*	Dummy variable. A value of 0 suggests an agricultural Hukou (rural registration), and a value of 1 impliess a non-agricultural Hukou (urban registration)
	*marry*	Dummy variable. A value of 0 indicates unmarried, and a value of 1 represents married / divorced / widowed
	*pension*	Dummy variable. 0 = not participating in pension insurance, 1 = participating in pension insurance
	*income*	Ordinal variable. A response of "very low" is assigned a value of 1; "average" is assigned a value of 2; "relatively high" is assigned a value of 3; "high" is assigned a value of 4; "very high" is assigned a value of 5
	*smoke*	Dummy variable. Smoking is assigned a value of 1, otherwise 0
	*exercise*	Dummy variable. Participating in physical exercise within the past month is given a value of 1, otherwise 0
	*noonbreak*	Dummy variable. Having a habit of taking a nap during the day is assigned a value of 1, otherwise 0

#### Calculation of digitalization levels

When measuring the digital literacy index, three methods are employed. In the baseline results, the entropy method is applied to objectively weight each indicator. The factor analysis method and scoring method are applied in the robustness checks.

1. Entropy method

It is assumed that there are m samples to be evaluated and n evaluation indicators, which form the original indicator data matrix $X = (x_{ij} )m \times n$, where $x_{ij} \ge 0$, $0 \le i \le m$,$0 \le j \le n$. For a certain indicator $x_{j}$, the larger the difference in the indicator $x_{ij}$ value, the greater role this indicator plays in the comprehensive evaluation. If the indicator values of a certain indicator are similar, then this indicator does not play a critical role in the comprehensive evaluation. In information theory, information entropy is $e_{j} = - k \cdot \sum\limits_{i = 1}^{m} {p_{ij} \cdot \ln p_{ij} }$, where $p_{ij} = x_{ij} /\sum\limits_{i = 1}^{m} {x_{ij} }$, $k> 0$.

The greater the variation in the value of a certain indicator, the smaller the information entropy, and the greater the amount of information provided by the indicator, which suggests a higher weight. Therefore, according to the degree of variation of each indicator, the information entropy is applied to calculate the weight of each indicator.

- Step 1: Measuring the proportion of the i-th sample under the j-th indicator $p_{ij}$.

1 $$p_{ij} = x_{ij} /\sum\limits_{i = 1}^{m} {x_{ij} }$$

- Step 2: Calculating the entropy value of the j-th indicator.

2 $$e_{j} = - k \cdot \sum\limits_{i = 1}^{m} {p_{ij} \ln p_{ij} }$$

where $$k> 0$$, $$k = 1/\ln m$$, and $$0 \le e \le 1$$.

- Step 3: Calculating the utility value of each indicator $d_{j}$.

For a given j, the smaller the variation in $d_{j}$, the larger the entropy value $e_{j}$. When there is a significant variation in the values of a certain indicator among the samples, $e_{j}$ becomes small, indicating that this indicator is more valuable in comparing digital literacy; thus, its weight is greater. The formula for calculating the utility value is:

3 $$d_{j} = 1 - e_{j}$$

- Step 4: Calculating the weight $w_{j}$ of indicator $x_{j}$:

4 $$w_{j} = d_{j} /\sum\limits_{j = 1}^{n} {d_{j} } = \frac{{d_{j} }}{{\sum\limits_{j = 1}^{n} {d_{j} } }} = \frac{{1 - e_{j} }}{{\sum\limits_{j = 1}^{n} {(1 - e_{j} )} }}$$

- Step 5: Calculating the index for digital literacy:

5 $$digital_{i} = \sum\limits_{j = 1}^{n} {w_{j} p_{ij} }$$

(2) Factor analysis

Factor analysis is a method for calculating comprehensive evaluation scores by reducing a number of related variables into a few uncorrelated new common factors. By reducing dimensionality, it simplifies the complexity of problem analysis while retaining the information of the original variables to the greatest extent.

The steps to calculate the comprehensive score of digital using factor analysis are as follows:

- Step 1: Estimate the factor loading matrix based on the original variable matrix. This study chooses the principal component method to estimate the factor loading matrix.

Assuming that the original indicator data matrix is $X = (x_{ij} )m \times n$, the covariance matrix of X is denoted as $\sum {}$.$\lambda_{1} \ge \lambda_{2} \ge \cdots \ge \lambda_{n}> 0$ represents the eigenvalue of $\sum {}$, and $\lambda_{i}$ represents the variance of principal component i. The total variance is specified as $\sum\limits_{i = 1}^{n} {\sigma_{ii} } = \sum\limits_{i = 1}^{n} {\lambda_{i} }$. $e_{1} ,e_{2} , \cdots ,e_{n}$ is the corresponding normalized orthogonal eigenvector. Therefore, $\sum {}$ can be decomposed as:

6 $$\begin{gathered} \sum { = \lambda_{1} } e_{1} e_{1}^{\prime } + \lambda_{2} e_{2} e_{2}^{\prime } + \cdots + \lambda_{n} e_{n} e_{n}^{\prime } \hfill \\ \begin{array}{*{20}c} {} & = \\ \end{array} (\sqrt {\lambda_{1} } e_{1} ,\sqrt {\lambda_{2} } e_{2} , \cdots \sqrt {\lambda_{n} } e_{n} )\left[ {\begin{array}{*{20}c} {\sqrt {\lambda_{1} } e_{1}^{\prime } } \\ {\sqrt {\lambda_{2} } e_{2}^{\prime } } \\ \vdots \\ {\sqrt {\lambda_{n} } e_{n}^{\prime } } \\ \end{array} } \right] \hfill \\ \end{gathered}$$

The decomposition in the above formula represents the covariance matrix structure of a factor model where the number of common factors is the same as the number of variables. When using the factor analysis methods, it is more preferable to reduce the number of common factors k until it is less than the number of variables, i.e., k < n. When the last n-k eigenvalues are relatively small, the contribution of the last n-k terms to $\sum {}$ is usually omitted, leading to:

7 $$\sum \approx (\sqrt {\lambda_{1} } e_{1} ,\sqrt {\lambda_{2} } e_{2} , \cdots \sqrt {\lambda_{k} } e_{k} )\left[ {\begin{array}{*{20}c} {\sqrt {\lambda_{1} } e_{1}^{\prime } } \\ {\sqrt {\lambda_{2} } e_{2}^{\prime } } \\ \vdots \\ {\sqrt {\lambda_{k} } e_{k}^{\prime } } \\ \end{array} } \right]$$

where $\sqrt {\lambda_{j} } e_{j}$ denotes the factor loading of common factor j.

- Step 2: Display the common factors as linear combinations of the variables to obtain the scores of each common factor. Since the number of equations k in the factor score function is less than the number of variables n, it is not possible to calculate the factor scores accurately. The factor scores can be estimated using the least squares method or the maximum likelihood method:

8 $$\hat{F}_{ij} = \beta_{i0} + \beta_{i1} x_{1} + \beta_{i2} x_{2} + \cdots + \beta_{in} x_{n}$$

- Step 3: Establish a comprehensive factor score function by weighted summation, using the proportion of each common factor's variance contribution rate to the total variance contribution rate of all the common factors as the weight:

9 $${Y}_{j }= {\gamma }_{1}{\widehat{F}}_{1j}+ {\gamma }_{2}{\widehat{F}}_{2j}+\cdots +{\gamma }_{k}{\widehat{F}}_{kj}, j=\text{1,2},\dots m$$

where $Y_{j}$ denotes the comprehensive factor score of sample j, $\hat{F}_{ij}$ represents the score of sample j achieved on common factor I, $\gamma_{i}$ is the proportion of common factor i's variance contribution rate to the total variance contribution rate, and $\gamma_{i} = \lambda_{i} /\sum\limits_{i = 1}^{n} {\lambda_{i} }$.

(3) Scoring methods

The scoring method is based on the respondents' answers to five questions regarding whether they access the Internet on a computer, whether they access the Internet on a mobile device, whether they make online purchases, whether they engage in online learning, and whether they participate in online entertainment. If the respondent answers "yes" to any of these questions, they receive 1 point. The scores for all five questions are then accumulated to obtain the digital literacy level of the senior citizens.

Based on the CFPS data from 2016, 2018, and 2020, this study constructs a balanced panel and employs a two-way fixed-effects model for the estimation. Based on the definition of WHO [60], the sample involved in this study comprised citizens aged 60 and above. After data cleaning and the removal of samples with missing values, the balanced panel data comprised 7836 samples, with 2612 samples per year. The descriptive statistics of the data are presented in Table 3.

**Table 3:** Descriptive statistics of variables

Variable	Mean	Std. Dev	Min	Max
*health*	2.5937	1.2163	1	5
*digital*	0.0780	0.1980	0	1
*issupport*	22.7985	4.4909	3	31
*iv1*	22.3355	44.3550	0	410.28
*iv1*	76.8740	197.5831	0	3688.66
*age*	67.8666	5.0804	60	93
*gender*	0.5398	0.4984	0	1
*edu*	4.2135	4.5865	0	16
*hk*	0.3382	0.4731	0	1
*marry*	0.8581	0.3490	0	1
*pension*	0.6770	0.4677	0	1
*income*	2.9543	1.1635	1	5
*smoke*	0.2985	0.4576	0	1
*exercise*	0.4856	0.4998	0	1
*noonbreak*	0.6605	0.4736	0	1

### Model

First, in order to examine the impact of digital literacy on the health status of senior citizens, a balanced panel model is established. The model is specified below:

10 $$health_{it} = \beta_{0} + \beta_{1} digital_{it} + \beta_{3} X_{it} + c_{i} + \theta_{t} + \varepsilon_{it}$$

where $health_{it}$ denotes the health status of senior citizen i in year t, $digital_{it}$ represents the digital literacy level of the senior citizen i in year t, $X_{it}$ is a series of control variables, $c_{i}$ and $\theta_{t}$ are individual and time fixed effects, and $\varepsilon_{it}$ stands for the random error term.

Next, to identify the mediating role of social support, the following models are constructed:

11 $$issurpport_{it} = \alpha_{0} + \alpha_{1} digital_{it} + \alpha_{3} X_{it} + c_{i} + \theta_{t} + \mu_{it}$$

12 $$health_{it} = \lambda_{0} + \lambda_{1} issurpport_{it} + \lambda_{2} digital_{it} + \lambda_{3} X_{it} + c_{i} + \theta_{t} + \eta_{it}$$

In models (11) and (12), $\text {issurpport}_{it\ it}$ stands for the intensity of social support. Coefficient $\lambda_{1}$ measures the impact of digital literacy on the health status of the senior citizens.

## Empirical results and discussions

### Baseline results

Table 4 reports the estimation results of the mixed effect models and the fixed effect models, and both show that digital literacy has a significant positive impact on the health status of senior citizens. According to the results from Column m4.4, after controlling for individual fixed effects, time fixed effects, and other control variables, t digital literacy significantly drives the health status of the elderly at the 5% level. Specifically, for every one-unit increase in the digital literacy index, the health status of the elderly improves by an average of 0.236 units, suggesting that an increase in digital literacy is beneficial to the health of the elderly, thus validating H1.

**Table 4:** Baseline results

*health*	Mixed effect model		Fixed effect model	
	m4.1	m4.2	m4.3	m4.4
*digital*	0.213^**^	0.188^**^	0.248^**^	0.236^**^
	(2.231)	(2.242)	(2.345)	(2.220)
*age*		−0.007^**^		−0.071^**^
		(−2.340)		(−2.261)
*gender*		0.176^***^		0.096
		(5.430)		(0.18)
*edu*		0.009^**^		0.014*
		(2.18)		(1.862)
*hk*		−0.0957^***^		−0.0102^**^
		(−2.85)		(−2.12)
*marry*		−0.0524		−0.048
		(−1.32)		(−0.52)
*pension*		0.058^**^		0.053^*^
		(1.970)		(1.844)
*income*		0.179^***^		0.0610^***^
		(15.30)		(4.926)
*smoke*		0.106^***^		0.179^***^
		(3.102)		(2.790)
*exercise*		0.119^***^		0.047^***^
		(4.196)		(3.612)
*noonbreak*		0.0445^**^		0.0703^**^
		(2.124)		(2.080)
*_cons*	2.585***	2.448^***^	2.574^***^	2.737^***^
	(175.071)	(12.492)	(200.898)	(18.688)
*Individual fixed effect*			Yes	Yes
*Time fixed effect*			Yes	Yes
*N*	7836	7836	7836	7836

^*^p < 0.1, ^**^p < 0.05, ^***^p < 0.01. *t*-values are reported in parentheses

### Endogeneity tests

The baseline result may be challenged by the endogeneity problems due to the omission of variables and the reversion causal relationship. Specifically, although econometric models can control for individual characteristic variables such as gender and age, there may still be factors that are difficult to observe or measure accurately, such as personal personality, preferences, and family culture. These unobservable variables that do not change over time cannot be identified in cross-sectional data, and instrumental variables are also powerless with regard to this issue, leading to endogenous problems caused by omitted variables. Moreover, elderly citizens who are in better health may have a higher likelihood of accessing the Internet and thus may enjoy greater convenience in obtaining digital information, potentially resulting in a higher level of digital literacy, leading to the issue of reverse causality.

This study tries to address endogeneity problems in three ways. First, this study constructs a balanced panel using the CFPS data from 2016, 2018, and 2020. In the baseline regression, this study controls for individual fixed effects and time fixed effects. The two-way fixed effects model can overcome the limitations of cross-sectional data and, to a certain extent, address the endogeneity issues arising from unobservable variables that do not change over time.

Second, this study employs the time-lagged explanatory variable for estimation. As shown in Column m5.1 in Table 5, the estimated coefficient for L.digital is significantly positive at the 5% level, which is consistent with the baseline regression.

**Table 5:** Results addressing endogeneity issues

	Lagged independent variables	IV estimation	
	m5.1	m5.2	m5.3
*L.digital*	0.218^**^		
	(2.135)		
*digital*		0.191^**^	0.198^**^
		(2.015)	(2.221)
*Control variable*	Yes	Yes	Yes
*Individual fixed effect*	Yes	Yes	Yes
*Time fixed effect*	Yes	Yes	Yes
*First-stage coefficient*		0.003^***^	0.004^***^
		(46.945)	(35.162)
*P-value for underidentification test*		0.000	0.000
*Weak-ID test (kleibergen-Paap Wald rk F statistic)*		2203.171	1235.922
*N*	5224	7836	7836

*p < 0.1, **p < 0.05, ***p < 0.01. *t*-values are reported in parentheses

Third, the instrumental variable (IV) method is employed for estimation. As mentioned above, this study employs the financial inclusion index (dfiindex) and the total postal and telecommunications business volume (tbtbusiness) to calculate the instrumental variables. The total postal and telecommunications business volume of provinces in 2008 and the digital inclusive finance index can reflect the level of digital infrastructure and the degree of digital transformation well; in turn, these affect the level of digital services enjoyed by the elderly and, consequently, their digital literacy levels. A higher total postal and telecommunications business volume in early years indicates a more developed postal and telecommunications industry and better facilities, leading to better current digital infrastructure and environment, which are conducive to improving the digital literacy of the elderly. Meanwhile, as an early macroeconomic variable, the total postal and telecommunications business volume of provinces in 2008 is unlikely to influence their current health. The health status of the elderly from 2016 to 2020 cannot affect the early postal and telecommunications business volume. Therefore, tbtbusiness satisfies the requirements of relevance and exogeneity as an instrumental variable.

The digital inclusive finance index published by Peking University is a scientific indicator for measuring the digital environment of a region [61]. A higher level of digital inclusive finance in an earlier period makes it easier for the elderly to access digital technologies and share digital benefits. However, the current health status of the elderly cannot affect the previous digital inclusive finance index; so, the previous dfiindex also satisfies the requirements of relevance and exogeneity as an instrumental variable.

When employing the IV method for estimation, in order to take into account micro-heterogeneity, the community average of digital literacy (mean_di) is introduced. Consequently, two instrumental variables are constructed. IV1 is the product of dfiindex and mean_di, while IV2 is the product of tbtbusiness and mean_di.

The results of the 2SLS estimation using the instrumental variable method are listed in Column m5.2 and Column m5.3 of Table 5. The first-stage estimation results indicate a strong positive correlation between the instrumental variable and digital literacy, suggesting that the better the digital environment, the higher the level of digital literacy, supporting the selection of the IV. The F-value of the weak-ID test is much greater than the critical value, further demonstrating the effectiveness of the IV. As can be seen from the results, the estimated coefficients for digital are positively significant at 5%, again supporting the findings of the baseline regression. Therefore, increasing the digitalization level is beneficial to the health of the elderly.

### Robustness tests

This study performs robustness tests from two aspects. First, the explanatory variables are replaced. We adopt both the scoring method and factor analysis method to calculate the digital literacy level among the elderly. In the scoring method, the elderly respondent’s digital literacy is determined based on their responses to five questions regarding whether they access the Internet on a computer, whether they access the Internet on a mobile device, whether they shop online, whether they learn online, and whether they engage in online entertainment. For each question, 1 point is received if the answer is "yes". Therefore, the total score from these five questions represents the elderly respondents’ digital literacy. The results are presented in Column m6.1 in Table 6. The specific indicators used to calculate the digitalization level index with factor analysis are the same as those used in the entropy method mentioned above. The results are reported in Column m6.2 in Table 6. The estimation results from both replacements are consistent with the baseline regression, indicating that the estimation results are robust.

**Table 6:** Results for robustness checks

	Replacing the explanatory variables		Replacing the dependent variable	
	m6.1	m6.2	m6.3	m6.4
*digital (scoring method)*	0.203^**^			
	(2.022)			
*digital (factor analysis)*		0.212^**^		
		(2.154)		
*digital*			0.196^**^	0.186^**^
			(2.110)	(2.078)
*Control variables*	Yes	Yes	Yes	Yes
*Individual fixed effect*	Yes	Yes	Yes	Yes
*Time fixed effect*	Yes	Yes	Yes	Yes
*N*	7836	7836	7836	7836

*p < 0.1, **p < 0.05, ***p < 0.01. *t*-values are reported in parentheses

Second, the dependent variable is replaced. We adopt the respondents' answers to the question "Have you felt physically unwell in the past two weeks?" in the questionnaire to replace the health status variable. The estimation results are shown in Column m6.3 of Table 6. The coefficient of digital remains positive and statistically significant. Moreover, CFPS also investigates citizens aged over 45 using seven questions regarding whether they can go out for outdoor activities independently, whether they can eat meals independently, whether they can perform kitchen activities independently, whether they can use public transportation independently, whether they can shop independently, whether they can clean and perform hygiene activities independently, and whether they can wash clothes independently. This study adopts a scoring method, where 1 point is given if the respondent answers "yes" to a certain question. The scores of these seven questions are summed up to replace the original dependent variable, and the re-estimated model is shown in Column m6.4 of Table 6. The estimation results remain consistent with the baseline regression results, again verifying H1.

### Mechanism analysis

The results above demonstrate that digital literacy promotes the health status of senior citizens. Does social support act as a mediator in the relationship between digital literacy and the health status of senior citizens? In order to identify the mediation mechanism, models (2) and (3) are estimated.

Table 7 displays the mediating results of social support. Column m7.1 displays the impact of digital on issupport, and m7.2 shows the impact of digital and issupport on health. These two columns together reveal the mediating impact of social support on the relationship between digital literacy and the health of the elderly. The estimated coefficients of digital and issupport are strongly significant. Moreover, the direct effect of digital on health in column m7.2 (0.219) is lower than the total effect observed in column m4.4 of Table 4 (0.236). Therefore, social support is a partial mediator in the relationship between digital literacy and health status. The degree of the mediating effect is the product of the coefficients $\alpha_{1}$ in Model (2) and $\lambda_{1}$ in Model (3), which is 0.017, accounting for 7.2% of the total effect. This result validates H2. That is, digital literacy improves the health status of senior citizens by enhancing social support.

**Table 7:** Results for mechanism analysis

	Depvar: *issupport*	Depvar: *health*
	m7.1	m7.2
*digital*	0.809^***^	0.219^**^
	(2.731)	(2.145)
*issupport*		0.021^**^
		(2.012)
*Control variables*	Yes	Yes
*Individual fixed effect*	Yes	Yes
*Time fixed effect*	Yes	Yes
*N*	7836	7836

*p < 0.1, **p < 0.05, ***p < 0.01. *t*-values are reported in parentheses

### Heterogeneous analysis

Since the impact of digital literacy on the health status of the elderly may vary among different groups, this study analyzes the heterogeneity of the impact from three perspectives: age, urban–rural type, and education level.

#### Age heterogeneity

In this section, the respondents are divided into younger elderly (aged 60–69) and middle-aged and older elderly (aged 70 and above). Based on the estimation results in Columns m8.1 and m8.2 of Table 8, the coefficient of digital is significantly positive only in the sample of younger elderly, indicating that the impact of digital literacy on the health status of senior citizens exhibits age heterogeneity.

**Table 8:** Heterogeneity results

	Age		Urban–rural type		Education level	
	60–69	70 and above	Urban	rural	non-illiterate	illiterate
	m8.1	m8.2	m8.3	m8.4	m8.5	m8.6
digital	0.285^**^	−0.026	0.347^**^	0.122^**^	0.371^**^	0.092
	(2.261)	(−0.102)	(2.115)	(2.001)	(1.988)	(1.125)
Control variables	Yes	Yes	Yes	Yes	Yes	Yes
Individual fixed effect	Yes	Yes	Yes	Yes	Yes	Yes
Time fixed effect	Yes	Yes	Yes	Yes	Yes	Yes
N	5315	2521	2650	5186	3881	3955

*p < 0.1, **p < 0.05, ***p < 0.01. *t*-values are reported in parentheses

#### Urban–rural heterogeneity

In this section, the sample is divided into urban and rural subsamples based on respondent location to analyze the urban–rural differences in the impact of digital literacy on the health status of the elderly. The estimation results in Columns m8.3 and m8.4 of Table 8 show that digital literacy has a significant driving effect on the health of both the urban and rural elderly, with the impact among the urban elderly being stronger.

#### Education level heterogeneity

In this section, the sample is divided into illiterate and non-illiterate groups based on respondent education level. As shown in Columns m8.5 and m8.6 of Table 8, the estimated coefficient of *digital* for the illiterate elderly is not significant, while it is significantly positive for the non-illiterate elderly.

## Discussions

This study utilizes the multi-period data from the CFPS database and examine the impact of digital literacy on the health status of senior citizens between 2016 and 2020. The entropy method is employed to measure the digital literacy levels of the elderly. A two-way fixed effects model is constructed, and the instrumental variable method is adopted for estimation to mitigate issues of reverse causality and omitted variable bias. Additionally, social support is introduced as the mediator, aiming to explore the mechanism through which digital literacy affects the health status of the elderly. This study seeks to provide Chinese evidence for the high-quality development of both the digital society and aging society, offering insights for developing countries in addressing related issues.

### The impact of digital literacy on the health status of senior citizens

It is found in the benchmark results that digital literacy contributes to the improvement of elderly health status. This conclusion remains valid in the endogeneity and robustness checks. H1 is therefore validated. This finding is consistent with those of Rajkhowa and Qaim [59] and Jin and Zhao [62], who found that a higher digital literacy level enables older people to access health information, enrich their leisure activities, and broaden communication channels with relatives and friends, which contribute to the improvement of their health status. Unlike their studies, we adopt an index system and the entropy weight method to measure digital literacy.

### The mediating effect of social support

In the mechanism analysis, it is found that digital literacy improves the health status of senior citizens through the channel of social support. H2 is verified. Existing literature found that income levels [29–32] and online entertainment [33] mediate the relationship between digital literacy and health. Our research reveals that social support also serves as a mediator between digital literacy and the health status of the elderly, thereby deepening the understanding of the influencing channels.

The possible explanation is that, digital knowledge practices have led to the reconstruction of traditional social relationships. Specifically, digital behaviors such as liking, sharing, and commenting have expanded the communication methods between the elderly and their peers [63]. This makes it easier for the elderly to form weak social ties with online acquaintances. Consequently, once equipped with a certain level of digital literacy, the social networks of older adults are no longer confined to traditional acquaintance circles [64]. Moreover, digital platforms bring together individuals with similar interests and hobbies, breaking existing social inertia and facilitating the elderly to obtain more social support [65]. Additionally, the digital-related interaction among families, such as children and grandchildren assisting older generations in enhancing their digital literacy, fosters intergenerational harmony and enhances mutual assistance and support. This will strengthen emotional bonds and psychological compensation, thereby enhancing the resilience and adaptability of family relationships [66].

### The heterogeneous impact of digital literacy on the health status of senior citizens

In the heterogeneous analysis, we found that the effect of digital literacy on the health status of senior citizens is stronger among the younger age group. For the younger elderly, their technical and learning barriers to digitalization are relatively low, resulting in a more frequent use of digital tools and better connection with social issues. However, as they become older, their cognitive abilities gradually decline, posing greater difficulties in using digital technologies. Therefore, the resulting health improvement effects are limited.

In addition, the effect of digital literacy on the health status of senior citizens is more pronounced in the urban elderly. This difference may stem from China's long-standing dual structure [67], where the aging process is more rapid, and the number of disabled elderly is higher in rural areas. Moreover, rural digital infrastructure significantly lags behind that of urban areas, leading to lower digital literacy levels among the rural elderly and resulting in a weaker health improvement effect.

Furthermore, the effect of digital literacy on the health status of senior citizens is stronger among the non-illiterate group. A possible explanation is that the illiterate elderly, due to their limited educational background, face challenges in learning and utilizing digital technologies, making it difficult for them to embrace the convenience and entertainment brought by digital devices. In contrast, the non-illiterate elderly possess sufficient knowledge and stronger learning abilities; so, the positive health effects of digital literacy are more pronounced in this group [68].

### Limitations

This study examines the impact of digital literacy on the health status of senior citizens and identifies the mediation mechanism, which enhances our understanding of the positive effects of digitalization on aging society. Meanwhile, there is still room for further improvements. First, the measurement of health status is a complicated process. Due to data availability, this study employs self-rated questions to measure the health status of the elderly, which may not be as accurate as clinical diagnostic instruments. Future research could be improved by obtaining elderly physical examination data for more precise measurements. In addition, we will try to further explore the availability of relevant resident health databases. By constructing a comprehensive health measurement index that incorporates multiple dimensions, such as physical examination results and self-rated health, future study can reduce the subjective bias of relying solely on self-rated health, enabling us to reveal the health levels of the elderly more comprehensively and accurately.

Secondly, this study focuses on the impact of digital literacy on the health status of the elderly, without further discussing its influence on health inequalities. The current research has not reached a consensus regarding whether digitization contributes to narrowing health inequalities among the elderly. That is, whether the development of digitization can assist the most disadvantaged individuals and reduce health inequalities remains a valuable research question and belongs to one of our next research efforts. In future research, we will try to classify senior citizens according to their digital literacy levels and measure the differences in health distribution across different digital literacy levels. The concentration index decomposition method will be employed to uncover the impact and mechanisms of digital literacy on health inequality of the elderly. This will provide insights for optimizing the medical security system in aging countries and bridging health disparities.

Lastly, this study utilizes micro-survey data, mainly discussing the impact of individual-level digital literacy at the micro level. Expanding our perspective to the macro level and discussing the health impact of digitization on the elderly would enhance the comprehensiveness of our research. In the future, we will incorporate comparative analyses across different regions and countries, discussing the impact of digitization on the health of the elderly from the perspective of human society.

## Conclusions and policy implications

Under the background of Healthy Aging and Digital China, it is of great significance to ensure that the elderly can enjoy the dividends of digitalization. Given this, digital literacy plays a crucial role in facilitating healthy aging among the elderly and promoting the Digital China strategy. This study discusses the theoretical mechanisms through which digital literacy affects the health status of senior citizens. Then, using the CFPS data in 2016, 2018, and 2020, this study empirically examines the impact of digital literacy on the health status of senior citizens.

The theoretical contributions are below. First, this study considers the elderly population as the research subject and explores the impact mechanism of digital literacy on the health status of senior citizens from both theoretical and empirical perspectives. Since the health of the elderly is a critical component of residential welfare, our study enables the formulation of effective policy recommendations tailored to China's current digital development from a health perspective. Our findings also serve as a scientific reference for other developing countries in their endeavors to promote the health status of senior citizens. Second, this study examines the potential channels through which digital literacy affects the health of the elderly from the perspective of social support. This not only narrows the gaps in the existing research about the micro impact of digitalization, but also provides insights for the health improvement of the elderly.

The main conclusions are as follows.

- First, digital literacy can improve the health status of senior citizens. This result remains valid after introducing lagged explanatory variables and addressing the endogeneity issues. The robustness of this finding is further confirmed through estimations with alternative explanatory and explained variables.
- Second, social support acts as a partial mediator in the relationship between digital literacy and the health status of senior citizens. An enhanced digital literacy level strengthens social support, thereby promoting the health status of the elderly.
- Third, the heterogeneity analysis reveals that the effect of digital literacy on the health status of senior citizens varies across age groups, urban–rural types, and education levels. Specifically, the positive effects of digital literacy are more pronounced among the younger, urban, and non-illiterate elderly.

Based on the above conclusions, the following policy implications are proposed.

First, joint efforts from multiple stakeholders should be made to release the positive impact of digital literacy on senior citizens’ health status. Governments should incorporate the improvement of digital literacy among the elderly into national strategies and encourage participation from all sectors of society in digital literacy training for the elderly to ensure their adaptability to life in the digital age. Additionally, efforts should be strengthened in constructing new types of digital infrastructure, such as big data platforms, to facilitate elderly individuals' access to data elements at a lower cost, thereby improving their digital literacy. At the societal level, greater attention should be paid to the internet usage of the elderly. For example, digital service volunteer stations specifically for the elderly should be established to address the challenges they face in using mobile devices such as smartphones. Community learning classes for smart devices should also be designed for the elderly. Furthermore, separate digital channels should be provided for the elderly in public places such as shopping malls and hospitals. Moreover, every citizen should lend a helping hand to the elderly in their vicinity regarding the use of digital tools.

Second, to broaden the way of elderly individuals to utilize digital tools to obtain social support, companies can further promote the elderly-friendly transformation of digital products, such as the artificial intelligence functions in smart home appliances. Enterprises can design more "elderly-friendly" interactive interfaces and functions, such as the "senior mode" with a comfortable experience, body control, and voice search, tailored to the behavioral habits of the elderly, ensuring that they can handle digital services without barriers. This will enable the elderly to gain social support more easily, thereby improving their health.

Third, the impact of digital literacy on the health of elderly individuals is relatively weak among the higher age group, those residing in rural areas, and those with lower levels of education. Therefore, it is imperative to focus on the cultivation of their digital literacy. Educational institutions should explore the establishment of a hierarchical lifelong education system regarding digital literacy among the elderly, such as the "Silver Age Digital Classroom" and other digital technology application teaching activities. In addition, the introduction of "new rural elites" serves as an effective means to improve digital literacy in rural areas. For instance, some returning college students and veterans utilize digital technology for daily use or entrepreneurship, thereby assisting or motivating elderly groups to adopt digital technology and acquire digital skills. Moreover, family members are encouraged to strengthen the cultivation of digital literacy among the less educated elderly through methods such as "digital feedback" and "intergenerational integration," enabling them to enjoy the health-promoting effects of digital literacy.

## Data Availability

The datasets supporting the study are publicly available on the CFPS website http://www.isss.pku.edu.cn/cfps/. The specific situation has been explained in the text. The raw data supporting the conclusions of this article will be made available by the authors upon request.

## Footnotes

1 Relevant data can be found at: https://www.stats.gov.cn/sj/zxfb/202402/t20240228_1947915.html.
